# Dietary Flavone Baicalein Combinate with Genipin Attenuates Inflammation Stimulated by Lipopolysaccharide in RAW264.7 Cells or *Pseudomonas aeruginosa* in Mice via Regulating the Expression and Phosphorylation of AKT

**DOI:** 10.3390/nu13124462

**Published:** 2021-12-14

**Authors:** Man Zhang, Lili Ye, Chuanjing Cheng, Fukui Shen, Lin Niu, Yuanyuan Hou, Gang Bai

**Affiliations:** 1 State Key Laboratory of Medicinal Chemical Biology, College of Pharmacy and Tianjin Key Laboratory of Molecular Drug Research, Nankai University, Haihe Education Park, 38 Tongyan Road, Tianjin 300353, China; 18202572363@163.com (M.Z.); ylili_ly1@163.com (L.Y.); 15522085313@163.com (C.C.); fukuishen@126.com (F.S.); houyy@nankai.edu.cn (Y.H.); 2 Laboratory of Compound Drugs and Systems Biology, Tianjin University of Traditional Chinese Medicine, Tianjin 300193, China; niulinnl@163.com

**Keywords:** baicalein, genipin, heat shock protein 90, AKT, synergistic anti-inflammatory effect

## Abstract

Mounting evidence has shown that single-targeted therapy might be inadequate to achieve satisfactory effects. Thus, drug combinations are gaining attention as they can regulate multiple targets to obtain more beneficial effects. Heat shock protein 90 (HSP90) is a molecular chaperone that assists the protein assembly and folding of client proteins and maintains their stability. Interfering with the interaction between HSP90 and its client proteins by inhibiting the latter’s activity may offer a new approach toward combination therapy. The HSP90 client protein AKT plays an important role in the inflammatory response syndrome caused by infections. In this study, the dietary flavone baicalein was identified as a novel inhibitor of HSP90 that targeted the N-terminal ATP binding pocket of HSP90 and hindered the chaperone cycle, resulting in AKT degradation. Combining baicalein with genipin, which was extracted from *Gardenia jasminoides*, could inhibit the pleckstrin homology domain of AKT, significantly increasing the anti-inflammatory effects both in vitro and in vivo. This synergistic effect was attributed to the reduction in AKT expression and phosphorylation. Thus, elucidating the mechanism underlying this effect will provide a new avenue for the clinical application and development of synergistic anti-inflammatory drugs.

## 1. Introduction

Cytokine storm (inflammatory storm) refers to the rapid and mass production of multiple cytokines in the body fluids post a microbial infection [1]. Patients with severe COVID-19 pneumonia develop evident cytokine storms compared to those with mild infection; the elevated cytokine levels may lead to deterioration or even death [2,3]. If a patient develops cytokine-storm-like complications, antiviral therapy alone is insufficient as a treatment, and anti-inflammatory medicine becomes necessary [4]. Inflammation is an inevitable pathophysiological process involving the interaction between injury and anti-injury factors [5]. Many studies have shown that inflammation is an essential immune response associated with the development of many diseases, including cancer and diabetes and those related to the cardiovascular system [6]. Various inflammation-related signal transduction pathways were identified, including the Keap1–Nrf2, TLR4–NF-κB, PI3K–AKT, JAK–STAT3, and MAPK pathways [7,8,9,10]. Among these, the anti-inflammatory response mediated by the PI3K–AKT signaling pathway plays a critical role in cells [11]. Many compounds were reported to regulate the PI3K–AKT pathway [12]. For example, glycyrrhizic acid exhibits significant anti-inflammatory effects by affecting the signal transduction in the PI3K–AKT signaling pathway and reducing cytokine production [13]. Additionally, andrographolide regulates glucose-induced damage by activating PI3K–AKT/iNOS signaling in human umbilical vein endothelial cells [14]. Studies have also shown that AKT can affect the expression of NF-κB-dependent chemokines, thereby regulating the transport of inflammatory cells [15]. Therefore, AKT is a key node of the PI3K/AKT signaling pathway and is a potential drug target for inflammatory regulation [16].

Drug combinations are widely used to treat various diseases, as they can enhance therapeutic effects, reduce side effects, and prevent drug resistance in patients [17]. For example, the combination of β-lactams and β-lactamase inhibitors plays an important role in fighting bacterial infections [18]. The combination of fludelone and panaxytriol, with its high efficiency and low toxicity, exerts a synergistic inhibitory effect on breast cancer progression [19]. Traditional medicines are more efficient than a single drug in treating chronic diseases. They target multiple channels and proteins; for example, the resins frankincense and myrrh exhibited excellent activity in inflammation and cancer therapy [20]. In previous research, the AKT inhibitors AZD6244 and GDC0914, which suppress the MEK–ERK and PI3K–AKT pathways, respectively, have been used in combination to cure aggressive thyroid cancers [21]. Different from the dual targeting of two pathways to regulate AKT and phosphorylation AKT, which may accompany certain side effects, better treatment strategies focused on synergistic AKT inhibition need to be developed to treat inflammatory diseases.

Scutellariae Radix and Gardeniae Fructus have been combined to treat infectious diseases for a long time as this combination is highly pharmacologically effective [22]. The dietary flavone baicalein (BAI) is one of the main components of *Scutellaria baicalensis* Georgi [23]. Many studies have reported that BAI exhibits several biological activities, including antioxidant, anti-inflammatory, antimicrobial, and antitumor activities [24,25,26,27]. Moreover, BAI suppresses cell proliferation, autophagy, and apoptosis in breast cancer cells. It also protects diabetic rats with cardiomyopathy from inflammation and oxidative-stress-induced damage and inhibits the growth of undifferentiated thyroid cancer cells [28,29,30]. Genipin (GNP), one of the main components of *Gardenia jasminoides* Ellis, is the product of hydrolysis of jasminoidin by β-glucosidase in vivo and was demonstrated to have significant anti-inflammatory activities [31]. It was reported that GNPs can activate the Nrf2 signaling pathway to inhibit lipopolysaccharide (LPS)-induced inflammatory response in BV2 microglia [31,32,33]. Moreover, studies have also shown that jasminoidin and BAI could act on BDNF and caspase-3 to treat stroke caused by cerebral ischemia-reperfusion injury [34].

Although both BAI and GNP were reported to exhibit significant anti-inflammatory effects, the mechanism underlying their synergistic effect is still unclear. This study synthesized a BAI molecular probe and identified HSP90 as its downstream target using chemical biology methods. Our results showed that BAI blocked the interaction between AKT and its chaperone protein HSP90, promoting the subsequent degradation of AKT. Subsequently, GNP was found to inhibit the phosphorylation of AKT. The synergistic relationship between BAI and GNP affected different aspects of AKT activity, inhibiting the PI3K–AKT signaling pathway and promoting anti-inflammatory effects, both in vitro and in vivo.

## 2. Materials and Methods

### 2.1. Materials

We obtained BAI and GNP (purity ≥98%, determined using HPLC) from Tianjin Heowns Biochemical Technology Co., Ltd. (Tianjin, China) and Shanghai YuanYe Biotechnology Co., Ltd. (Shanghai, China), respectively. The inducible nitric oxide synthase (iNOS) inhibitor S-methylisothiourea sulfate (SMT) was purchased from Beyotime Biotechnology (Shanghai, China). LPS and dexamethasone (DEX) were purchased from Sigma Corporation (St. Louis, MO, USA). Magnetic nanoparticles (MMs) were obtained from the Tianjin Baseline ChromTech Research Center (Tianjin, China). Adenosine triphosphate (ATP) and the HSP90 inhibitor geldanamycin (GEL) were purchased from MedChemExpress (Monmouth Junction, NJ, USA). Rabbit monoclonal iNOS, P-AKT (Ser473), polyclonal GAPDH, and Alexa-Fluor^®^-488-conjugated and -405-conjugated antibodies were obtained from Abcam (Cambridge, UK). Rabbit monoclonal HSP90, AKT, and secondary antibodies (goat anti-rabbit IgG H&L) were obtained from Cell Signaling Technology (Boston, MA, USA). Alexa-Fluor^®^-647-conjugated anti-mouse antibody was purchased from Thermo Fisher Scientific (Waltham, MA, USA). The biotin-conjugated mouse antibody P-AKT (Ser473) was purchased from Rockland Immunochemicals (Gilbertsville, PA, USA). Alexa-Fluor^®^-488-conjugated streptavidin and mouse polyclonal AKT antibodies were purchased from Bioss Inc. (Beijing, China). Protein A + G agarose was obtained from Bioworld (Minneapolis, MN, USA). *Pseudomonas aeruginosa* 14 strain (PA 14) was acquired from Associate Professor Bai Fang’s group at Nankai University (Tianjin, China).

### 2.2. Cell Culture

The RAW264.7 cell line was purchased from American Type Culture Collection (Rockville, MD, USA). The cells were cultured in Roswell Park Memorial Institute (RIPA)-1640 medium with 10% fetal bovine serum and 1% (unit/mL) penicillin/streptomycin at 37 °C in a 5% CO_2_ humidified atmosphere. We evaluated the cell viability via the CCK-8 Assay Kit (MedChemExpress), which was used as per the manufacturer’s instructions.

### 2.3. Target Fishing

To identify the targets of BAI, an alkynyl-modified BAI probe (BAI probe) was synthesized in advance, which was verified using NMR. ^1^H NMR (400 MHz, chloroform-d) δ 12.54 (s, 1H), 7.89 (dd, *J* = 7.9, 1.8 Hz, 2H), 7.53 (d, *J* = 7.4 Hz, 3H), 6.75 (s, 1H), 6.69 (s, 1H), 5.29 (s, 1H), 4.90 (d, *J* = 2.5 Hz, 2H), 2.63 (t, *J* = 2.4 Hz, 1H). ^13^C NMR (101 MHz, chloroform-d) δ 182.8, 164.457, 150.7, 150.4, 146.2, 132.0, 131.5, 130.2, 129.2, 126.4, 106.6, 105.5, 92.3, 77.3, 77.2, 57.1. Subsequently, we prepared BAI-probe-modified functionalized magnetic microspheres (BAI-MMs) using the click reaction, as previously described in [35]. We plated the RAW264.7 cells in 75 cm^2^ Petri dishes until the cell density reached 90%. The cells were washed with precooled PBS, following which, 600 μL of RIPA lysis buffer was added to the cells for 30 min. The lysates were collected and the protein solution was centrifuged for 10 min. Then, the supernatants of cell lysates and BAI-MMs were blended using a four-dimensional spinner at 4 °C for 12 h. Subsequently, the magnetic beads were eluted using a magnet and washed thrice with precooled PBS. DL-dithiothreitol (DTT; 500 μL, 100 μM) was added to the eluate, and the captured target proteins were released at 24 °C for 30 min. After evaluation using SDS-PAGE and western blotting, the captured proteins were identified using tandem mass spectrometry (MS/MS) ion protein profiling (Huada Protein R&D Center Co., Ltd., Beijing, China).

### 2.4. Target Prediction

Virtual docking of the three-dimensional structure of BAI (sdf format) was performed using PharmMapper (http://lilab.ecust.edu.cn/pharmmapper/, accessed on 15 June 2020). The potential targets of BAI were determined using a Venn diagram analysis that included the docking data obtained from PharmMapper, the proteins captured using BAI-MMs, and the anti-inflammatory proteins obtained from the GeneCards database (http://www.genecards.org/, accessed on 20 September 2020).

### 2.5. Microscale Thermophoresis (MST)

We labelled the HSP90 at a concentration of ~40 nM using the Monolith NT Protein Labeling Kit RED (Cat#L001, Nano-Temper Technologies, Notamp Technology Co., Ltd., Beijng, China), as per the manufacturer’s protocol. Subsequently, we titrated BAI against 39 μM of 5% (*v*/*v*) DMSO to obtain a 1:1 dilution. HSP90 proteins were expressed and purified according to our previous research [36]. Samples were diluted in PBS (pH 7.4) with 5% DMSO. Subsequently, the samples were placed in hydrophilic capillaries (Cat#K004) and analyzed using a Monolith NT.115 (NanoTemper, Biberach, Germany).

### 2.6. Fluorescence Quenching Assay (FQA)

The purified HSP90 (10 μM) and serially diluted BAI samples (0–100 μM) were mixed in equal proportions, and 100 μL/well of the resulting solution was added into a 96-well plate. A multifunctional microplate reader (TECAN, Spark) was used to measure the fluorescence of the tagged HSP90s at 37 °C. The excitation wavelength was set at 280 nm, and the emission wavelength ranged from 300–450 nm. Subsequently, the dissociation constant K_D_ between BAI and HSP90 was calculated.

### 2.7. Co-Immunoprecipitation

To evaluate the interaction between HSP90 and AKT in the presence or absence of BAI, a co-immunoprecipitation (Co-IP) assay was performed. We added BAI (10 μM) to the RAW264.7 cell lysates and incubated them overnight at 4 °C. Subsequently, 1 μL of rabbit AKT antibody was added to 50 μL of the cell lysate, and the resultant solution was incubated in a four-dimensional spinner at 4 °C for 8 h. Following this, 20 μL of protein A + G-agarose beads were added to the solution at 4 °C for 3 h. The beads were precipitated via centrifugation at 2500 rpm at 4 °C for 2 min, and then the supernatants were discarded. After washing with precooled PBS, the beads were boiled in an SDS buffer at 100 °C for 5 min. Eventually, the eluted proteins were evaluated by Western blotting.

### 2.8. Co-Localization Test

We divided the RAW264.7 cells into six groups: control, model, GEL (5 μM), BAI (8 μM), GNP (2 μM), and the combination group of BAI (8 μM) and GNP (2 μM). All groups, except the control group, were stimulated with LPS (100 ng/mL) for 24 h. Thereafter, the cells were fixed with 4% paraformaldehyde for 10 min and washed thrice with precooled PBS. The cells were blocked with 10% goat serum for 1 h and incubated with rabbit anti-HSP90 antibody (1:100) at 4 °C overnight. After washing with PBST, Alexa-Fluor^®^-405-conjugated goat anti-rabbit secondary antibody (1:500) was added to the cells and incubated for 2 h at 37 °C. Similarly, the cells were also incubated with mouse anti-AKT antibody (1:200) and Alexa-Fluor^®^-647-conjugated secondary antibodies (1:500). Subsequently, the cells were treated with an anti-AKT pS473 biotin antibody (1:500) and an AF-488 streptavidin secondary antibody (1:500). Eventually, images were obtained using a confocal fluorescence microscope (Leica TCS SP8).

### 2.9. Combination Index Analysis

RAW264.7 cells were seeded in 96-well plates till the cell density reached ~60%. Thereafter, the cells were treated with SMT (10 μM), BAI (5, 10, 20, 40, and 50 μM), GNP (5, 10, 20, 40, and 50 μM), or a combination of BAI (5, 10, 20, 40, and 50 μM) and GNP (1, 5, and 10 μM). The treated cells were incubated for 24 h in the presence of LPS (100 ng/mL). Subsequently, we determined the nitric oxide (NO) content using the NO kit (Biotechnology, Shanghai, China) as per the manufacturer’s instructions. The IC_50_ values of BAI and GNP administered alone or in combination with NO activity were calculated. To determine the synergistic effects of BAI and GNP, we calculated the combination index (CI) of their various concentrations and ratios in a combination using CompuSyn software. In brief, CI < 1.0 indicated a synergistic effect, CI = 1.0 indicated an additive effect, and CI > 1.0 indicated an antagonistic effect.

### 2.10. Western Blotting

RAW264.7 cells were cultured in six-well plates for 12 h with or without an LPS (100 ng/mL) treatment. The cells were then co-cultured with GEL, BAI, GNP, or the combination of BAI and GNP for 24 h. After washing with precooled PBS, the cells were lysed in 60 μL of RIPA lysis buffer and were incubated on ice for 30 min. Subsequently, we collected the lysates and separated the proteins in the lysate using SDS-PAGE and transferred them to a polyvinylidene fluoride membrane for Western blotting. The membranes were blocked with 5% skim milk at 24 °C for 2 h and then incubated overnight at 4 °C with the following primary rabbit antibodies: HSP90 (1:1000), AKT (1:1000), P-AKT^473^ (1:5000), iNOS (1:1000), and GAPDH (1:5000). The membranes were washed with PBST and incubated with goat anti-rabbit IgG H&L secondary antibody (1:3000) for 1 h at room temperature. Thereafter, the membranes were immersed in a chemiluminescent reagent and visualized using Tanon-5200.

### 2.11. Acute Lung Injury Test

Male Kunming mice (18–25 g) were obtained from the Vital River Laboratory Animal Technology Co. Ltd. (Beijing, China). All mice were freely provided with the same food and water and kept at 24 °C with 12 h light/12 h dark cycles. The mice were randomly divided into six groups (*n* = 6): control, model (PA 14), DEX (10 mg/kg/day), BAI (10 mg/kg/day), GNP (2.5 mg/kg/day), and the combination group (10 mg/kg/day BAI and 2.5 mg/kg/day GNP). The mice were administered with the drugs via intraperitoneal injection for one week. Following this, 10 μL of activated PA 14 strain (0.5 × 10^7^ in PBS) was administered into the nasal cavities of the mice to induce acute pulmonary infection [37]. Post 24 h, bronchoalveolar lavage fluid (BALF) was collected from the right lung for cytokine detection. Then, the lung tissues were soaked in normal saline and homogenated using an electric homogenizer, which were utilized for Western blotting. The middle lobes of the left lung were rinsed in a normal saline solution and fixed in 4% paraformaldehyde. Subsequently, lung tissue sections (embedded in paraffin) were stained using the hematoxylin and eosin stain for histomorphology or immunofluorescence.

### 2.12. Statistical Analysis

All data are expressed as mean ± standard deviation (SD). We used GraphPad Prism (version 8.0) for the statistical analysis. Significant differences were analyzed using the *t*-test between the two groups and by one-way ANOVA for those between multiple groups. A *p*-value < 0.05 was considered statistically significant.

## 3. Results

### 3.1. HSP90 as a Potential Target of BAI

**Figure 1:**
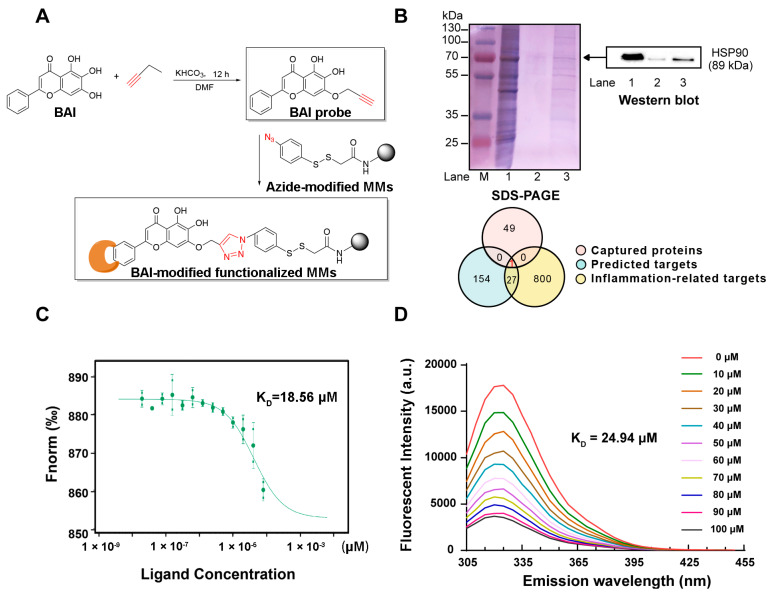
BAI targeted HSP90. (**A**) Synthesis of alkynyl-BAI (BAI probe) and its application in target fishing. (**B**) Identified protein targets of BAI. M: protein marker; lane 1: RAW264.7 cell lysates (loading control); lane 2: proteins captured by magnetic microspheres (negative control); lane 3: proteins captured by BAI probe modified magnetic microspheres. Integrated Venn diagram of captured proteins, predicted proteins, and anti-inflammatory proteins. (**C**) Interactions between BAI and HSP90 determined using MST. (**D**) The FQA assay of HSP90 proteins with BAI. K_D_ is the dissociation constant. BAI, baicalein; HSP90, heat shock protein 90; MMs, magnetic nanoparticles; MST, microscale thermophoresis.

We identified the potential target proteins of BAI in RAW264.7 cells by target fishing using BAI-MMs (Figure 1A). As depicted in Figure 1B (left panel), we observed that the BAI-MMs could capture more proteins (lane 3) than the negative group (lane 2). Subsequently, we identified 50 proteins with a protein profile score ≥100. Additionally, we integrated the data on the anti-inflammatory protein targets of BAI, obtained by virtual docking on PharmMapper (fit value ≥ 2, 182), with the inflammation associated proteins obtained from the GeneCards database (score > 7.8, 828) in a Venn diagram (Figure 1B). The intersection between these three datasets was demonstrated that HSP90 could be a BAI target. Additionally, this speculation was further verified using Western blotting (Figure 1B, right panel).

To further validate the interaction between HSP90 and BAI, we evaluated the binding properties using small-molecule and target-interaction assays. Our observations indicated that the binding affinity between HSP90 and BAI was ~18.56 μM, as measured using MST (Figure 1C). Furthermore, we observed that at 325 nm, the maximum emission wavelength, the endogenous fluorescence intensity of HSP90 weakened in a dose-dependent manner in response to increasing doses of BAI (0–100 μM; Figure 1D). Consequently, the K_D_ value was ~24.94 μM. Both these observations validated that BAI targeted HSP90.

### 3.2. BAI Induced AKT Degradation and Decreased P-AKT

**Figure 2:**
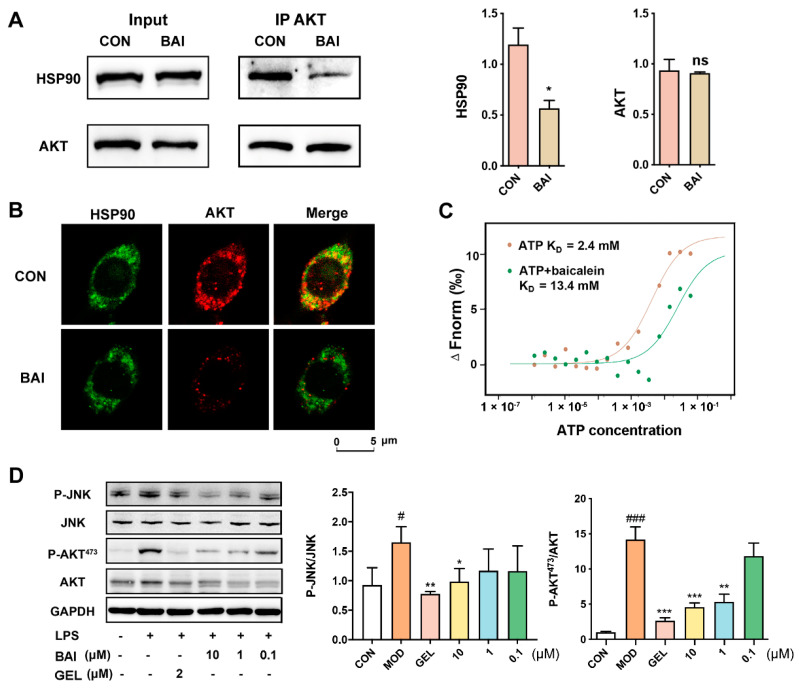
BAI bound to the ATP-binding domain of HSP90 to induce AKT degradation. (**A**) Co-IP of the HSP90 and AKT proteins with or without BAI (*n* = 3). * *p* < 0.05 vs. CON group. (**B**) Co-localization of HSP90 (pseudo-red) with AKT (pseudo-green) with or without 10 µM BAI administration in RAW264.7 cells. (**C**) BAI competing with ATP to target HSP90 measured using MST. (**D**) BAI reduced the expression of P-JNK and P-AKT stimulated using LPS in RAW264.7 cells in a concentration-dependent manner (*n* = 3). ^#^ *p* < 0.05, ^###^ *p* < 0.001 vs. CON group; * *p* < 0.05, ** *p* < 0.01 and *** *p* < 0.001 vs. MOD group; ATP, adenosine triphosphate; BAI, baicalein; CON, control; GEL, geldanamycin; HSP90, heat shock protein 90; LPS, lipopolysaccharide.

AKT is known to be a classic client protein of HSP90, and inhibiting the interaction of HSP90 with AKT can induce the ubiquitin-mediated degradation of AKT [38]. We elucidated the effect BAI had on the interaction between AKT and HSP90 using Co-IP. As depicted in Figure 2A, either HSP90 or AKT proteins could capture each other from the RAW264.7 cell lysate. Notably, post incubation with BAI, binding between the two proteins weakened, reducing the yield of precipitated proteins of HSP90. These observations suggested that BAI hindered the interaction between HSP90 and AKT. We validated this observation visually via the co-localization test. We observed that the pseudo-green fluorescence of HSP90 and the pseudo-red fluorescence of AKT merged to yellow in the absence of BAI (Figure 2B). However, the addition of BAI to the cells weakened the intensity of the merged yellow, suggesting that BAI affected the binding of HSP90 to AKT and reduced the relative concentration of AKT.

The N-terminal ATP-binding domain is considered to be the most important domain of HSP90 and is essential for maintaining its structure and function and for stabilizing the client proteins of HSP90 [39]. Since BAI is a potential inhibitor of HSP90, we speculated whether it interacted with HSP90 via the ATP-binding pocket. We evaluated whether BAI competed with ATP to bind to the ATP binding pocket of HSP90 by performing MST. We noted that the binding affinity of ATP with HSP90 was about 2.4 mM in the absence of BAI; however, it increased to 13.4 mM in the presence of BAI. This indicated that BAI potentially targeted the ATP-binding pocket of HSP90 (Figure 2C). To verify the effect of BAI on HSP90 and its client proteins, LPS was used to stimulate the downstream signaling effectors of HSP90 in RAW264.7 cells. P-JNK and P-AKT^473^ were selected here as the representative downstream effectors of HSP90. As shown in Figure 2D, the protein expression levels of both P-JNK and P-AKT^473^ were increased after LPS stimulation. GEL, an HSP90 inhibitor that targets the HSP90 ATPase binding site, acted as a positive control. BAI significantly prevented the activation of HSP90 in a concentration-dependent manner.

### 3.3. The CombInation of BAI and GNP Enhanced the Inhibition of AKT Activation

**Figure 3:**
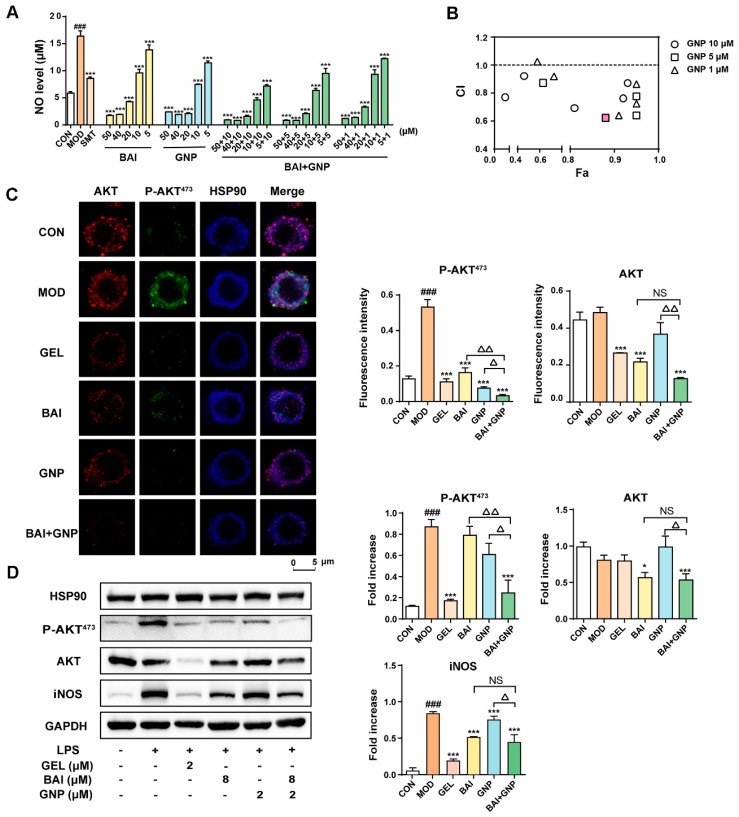
The combined anti-inflammatory effect of BAI and GNP in LPS-induced RAW264.7 cells. (**A**) Inhibitory levels of BAI and GNP on nitric oxide production in LPS-induced RAW264.7 cells. (**B**) Combination index between BAI and GNP. (**C**) The representative image of the combined effect of BAI and GNP on P-AKT and AKT detected using co-localization. (**D**) Expression of P-AKT^473^, AKT, and iNOS attenuated by the combination of BAI and GNP (*n* = 3). Each bar represents the mean ± SD. ^###^ *p* < 0.001 vs. CON group; * *p* < 0.05 and *** *p* < 0.001 vs. MOD group; ^△^ *p* < 0.05 and ^△△^ *p* < 0.01 vs. BAI + GNP group. BAI, baicalein; CON, control; GNP, genipin; GEL, geldanamycin; HSP90, heat shock protein 90; INOS, inducible nitric oxide synthase; LPS, lipopolysaccharide; MOD, model; NS, no significance; SMT, S-methylisothiourea sulfate.

Previous studies demonstrated that GNP acts on the pleckstrin homology (PH) domain of AKT and can inhibit the phosphorylation of AKT^473^ [40]. Thus, we hypothesized that the combination of BAI and GNP had the potential to induce a synergistic anti-inflammatory effect. We indirectly evaluated the combined effect of BAI and GNP on AKT by estimating the production of nitric oxide (NO), a downstream effector of AKT. We found that RAW264.7 cells stimulated with LPS showed significantly increased levels of NO. However, treatment with different concentrations of BAI (5–50 µM) and GNP (5–50 µM) significantly decreased the cellular NO levels. In addition, the combination of BAI and GNP had a more dramatic effect (Figure 3A). Our observations further indicated that BAI and GNP had a synergistic effect, and their optimal molar ratio was 4:1 (pink square shown in Figure 3B). Then, the combination of BAI (8 µM) and GNP (2 µM) was selected for use in the subsequent co-localization and AKT activation tests in the LPS-induced RAW264.7 cells.

As depicted in Figure 3C, the blue fluorescence intensity of HSP90 hardly changed throughout the co-localization experiment. Upon the induction of LPS in the RAW264.7 cells, the pseudo-green fluorescence of P-AKT^473^ in the model group increased significantly. However, when the cells were treated with BAI and GNP, the fluorescence intensity decreased by varying degrees, similar to our observations in the positive control GEL. Compared to the single-drug group, the combination group showed the most significant change in fluorescence intensity. The pseudo-red fluorescence intensities of AKT in the control, model, and GNP groups were similar to one another, whereas those in the GEL, BAI, and BAI + GNP groups were significantly reduced. In addition, our Western blotting results revealed that BAI reduced the expression of AKT and iNOS in the cells, while GNP did not. While the phosphorylation of AKT and expression of iNOS increased after LPS stimulation, the addition of BAI and GNP to the cells decreased the phosphorylation of AKT. As expected, the combined treatment had a more significant effect on decreasing the AKT protein level than other treatment approaches (Figure 3D).

**Figure 4:**
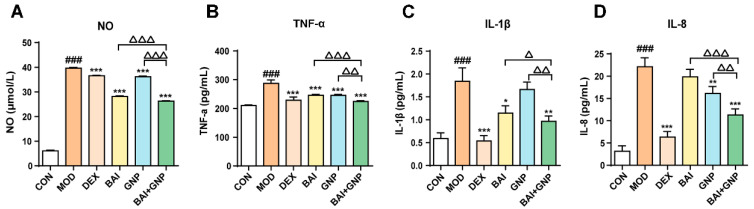
Lipopolysaccharide-induced synergistic inhibition of NO (**A**), TNF-α (**B**), IL-1β (**C**), and IL-8 (**D**) productions by BAI and GNP (*n* = 6). Each bar represents the mean ± SD. ^###^ *p* < 0.001 vs. control group; * *p* < 0.05, ** *p* < 0.01, and *** *p* < 0.001 vs. model group; ^△^ *p* < 0.05, ^△△^ *p* < 0.01, and ^△△△^ *p* < 0.001 vs. combination group. BAI, baicalein; CON, control; DEX, dexamethasone; GNP, genipin; MOD, model; NO, nitric oxide; IL-1β, interleukin 1β; IL-8, interleukin 8; TNF-α, tumor necrosis factor-α.

Based on the above results, we observed that BAI reduced the AKT phosphorylation by decreasing the amount of AKT in the cells; on the other hand, GNP directly inhibited the AKT phosphorylation. Hence, the combined treatment affected both the expression and phosphorylation of AKT, significantly improving the anti-inflammatory effect of the treatment and alleviating the inflammatory response mediated by NO, TNF-α, IL-1β, and IL-8, which were detected in the LPS-stimulated RAW264.7 cells (Figure 4A–D).

### 3.4. The Combination of BAI and GNP Alleviated Bacteria-Induced Acute Lung Injury in Mice

**Figure 5:**
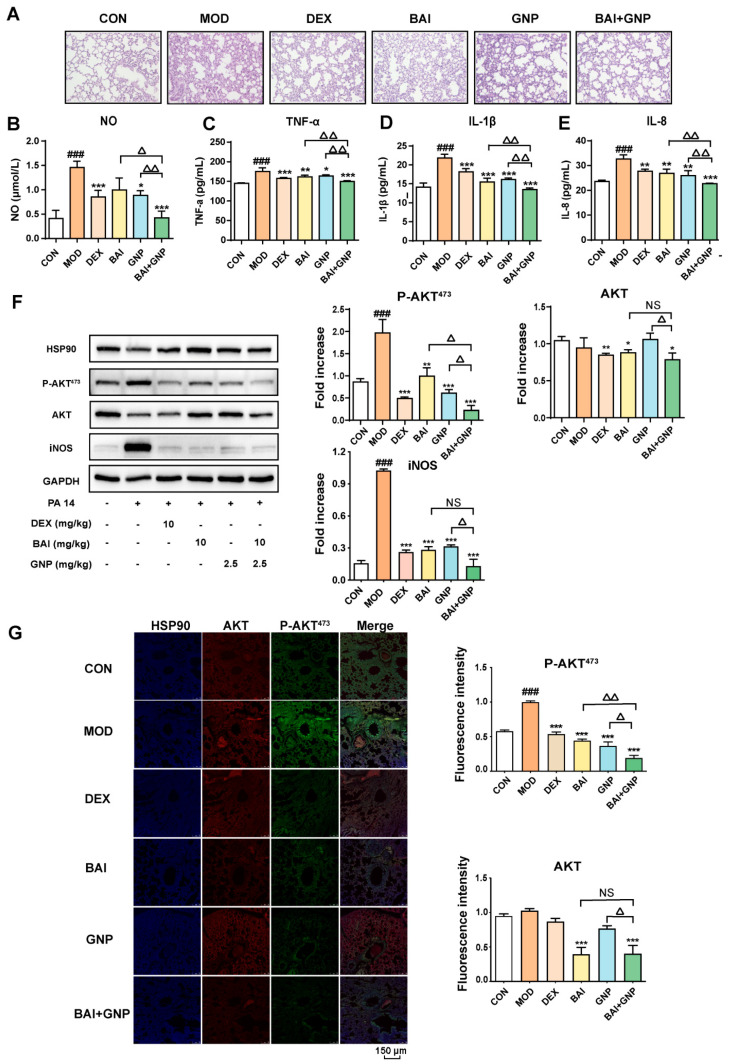
Synergistic effects of BAI and GNP on acute lung injury induced by the PA 14 bacterial strain in mice. (**A**) Representative images of hematoxylin and eosin staining of lung tissues (400×). (**B**–**E**) BAI and GNP synergistically inhibited the production of nitric oxide, TNF-α, IL-1β, and IL-8, respectively, induced by lipopolysaccharide in bronchoalveolar lavage fluid of mice (*n* = 6). (**F**) The combination of BAI and GNP attenuated the expression on P-AKT^473^, AKT, and iNOS (*n* = 3). (**G**) BAI and GNP synergistically decreased the protein levels of AKT and P-AKT^473^ in lung tissue (*n* = 3). Each bar represents the mean ± SD. ^###^ *p* < 0.001 vs. control group; * *p* < 0.05, ** *p* < 0.01, and *** *p* < 0.001 vs. model group; ^△^ *p* < 0.05 and ^△△^ *p* < 0.01 vs. combination group. BAI, baicalein; CON, control; DEX, dexamethasone; GNP, genipin; HSP90, heat shock protein 90; IL-1β, interleukin 1β; IL-8, interleukin 8; INOS, inducible nitric oxide synthase; MOD, model; NO, nitric oxide; PA 14, *Pseudomonas aeruginosa* 14 strain; TNF-α, tumor necrosis factor-α.

P. aeruginosa is an opportunistic pathogen that causes acute lung injury. To verify the combined effect of BAI and GNP in reducing inflammation in vivo, we used the PA 14 strain to induce acute lung injury in mice. Consequently, we observed a remarkable increase in neutrophil infiltration, alveolar hemorrhage, and peripheral angioedema and an increase in the thickness of the alveolar walls in the lung tissues of mice in the model group compared to that in the control group. However, BAI and GNP pre-administered to the mice alleviated these pathological changes to varying degrees; notably, this effect was the most prominent in mice belonging to the combination group (Figure 5A), similar to that observed in mice belonging to the DEX group. Subsequently, we estimated the cytotoxic factors or cytokines in the mouse BALF to evaluate the anti-inflammatory effect of the treatments. We noticed that treatment with both BAI and GNP decreased the levels of NO, TNF-α, IL-1β, and IL-8. Thus, the anti-inflammatory effect was more significant in the combination group than that in the other treatment groups (Figure 5B–E).

In addition, we analyzed the protein expression in all the groups and observed that the expression of AKT and iNOS in the DEX and BAI groups were lower than those in the model and GNP groups. This indicated that BAI reduced the levels of AKT in the lung tissue, whereas GNP did not. Moreover, phosphorylation of AKT in the lung tissue was observed to be significantly higher in the model group than that in the control group. We observed different degrees of decline in the protein level of P-AKT^473^ and iNOS treatment with BAI or GNP. Moreover, the administration of both BAI and GNP was more effective in reducing P-AKT^473^ than that of individual drugs (Figure 5F,G). This observation demonstrated that the combination of BAI and GNP enhanced the anti-inflammatory effect and could effectively improve acute lung injury in mice.

## 4. Discussion

Lung injury is a common ailment that is caused either directly by damage to the lungs or indirectly by an inflammatory process. A systemic inflammatory response can lead to acute lung injury and even death. Toll-like receptor 4 (TLR4) is the primary receptor for LPS-mediated immune activation [41,42], and the combination of TLR4 with LPS activates downstream signaling pathways, affects signal transduction, and promotes pro-inflammatory cytokine release [43]. A crucial protein downstream of TLR4 is AKT, which is involved in the LPS-induced immune response. It mediates signal transduction from a variety of upstream regulatory proteins (PTEN, PI3K, and tyrosine kinases) to downstream effectors (such as glycogen synthase kinase 3β, FOXO, and MDM2), and it mediates various biological functions [44]. Thus, regulating the AKT signaling pathway may significantly control LPS-induced inflammation. For example, dasatinib regulates LPS-induced microglial and astrocytic neuroinflammatory responses by inhibiting AKT/STAT3 signaling [45].

The chaperone protein HSP90 is a ubiquitous and highly conserved protein that supports accurate protein assembly and folding. It also maintains the stability of client proteins, including the functional maturity and stability of some signal proteins [46,47]. The structure of HSP90 consists of three major domains: an N-terminal domain (NTD) that possesses ATPase activity, harbors ATP and most drug-binding sites, and has a motif that regulates the folding of client proteins; a middle domain that recognizes and binds to client proteins and forms the active ATPase; and a C-terminal domain containing a dimerization motif [48]. Notably, AKT is one of the client proteins of HSP90 that exists as a complex with HSP90 in vivo. Moreover, HSP90 maintains the protein stability of AKT [49,50].

Most drugs target the N-terminal ATP-binding site of HSP90, which has a crucial ATPase activity. During the chaperone cycle, ATP binds to NTD and induces the rotation and dimerization of the NTDs of two HSP90s to form a “closed” conformation. This conformational change causes the dimerized HSP90s to be catalytically active, and then the bound ATP is hydrolyzed, releasing ADP and energy. Thereon, the conformation becomes more “closed” than before, and the client protein is folded simultaneously. Eventually, ADP dissociates and the conformation changes to an “open” state, releasing the folded client protein, and the next chaperone cycle is initiated [51]. Hence, preventing the binding of ATP to HSP90 can affect the function of the latter, thereby indirectly affecting the activity of AKT. Moreover, inhibition of HSP90 and AKT binding can exert anti-inflammatory effects. Sato et al. constructed deletion mutants of AKT and HSP90 and determined that amino acid residues 327–340 of HSP90 bind to AKT and amino acid residues 229–309 of AKT bind to HSP90 [52]. Furthermore, interfering with the interaction between these two proteins leads to the degradation of AKT, thereby inhibiting the downstream signal transduction of inflammation-inducing signals [53]. In this study, we found that BAI changed the conformation of HSP90 by targeting the ATP binding site in the NTD of HSP90. This prevented the interaction of HSP90 with the AKT complex, leading to ubiquitin-mediated degradation of AKT.

The protein AKT contains three domains: an N-terminal PH domain with a high affinity for phosphatidylinositol-3,4,5-triphosphate (PIP3), a conserved kinase domain, and a C-terminal regulatory domain [54]. The activation of AKT involves targeting the membrane protein phosphatidylinositol 3-kinase (PI3K). Upon receiving a growth-factor-mediated stimulus, PI3K is indirectly activated by activated receptor tyrosine kinases, leading to the generation of PIP3. Subsequently, AKT is recruited from the cytoplasm to the plasma membrane and binds to PIP3, and is activated by phosphorylation at Thr308 in the kinase domain and Ser473 in the C-terminal domain [49]. Based on the structure of AKT, its inhibitors are classified into three categories: allosteric inhibitors, ATP competitive inhibitors, and PH domain inhibitors. Phillygenin, the main chemical constituent of Forsythia suspensa, targets the allosteric inhibition pocket of AKT and inhibits the AKT downstream signaling pathways [55]. Ipatasertib (GDC-0068) is a novel small-molecule ATP-competitive inhibitor that prevents the phosphorylation of the client proteins downstream of AKT by competitively binding to the ATP-binding pocket [56]. Furthermore, GNP, the active ingredient in *Gardenia jasminoides*, targets the PH domain of AKT and affects AKT plasma transport, blocks the PI3K–AKT signaling pathway, and ultimately exerts an anti-inflammatory effect in the body [40].

Increasing attention has been paid to drug combinations in recent years. Combination therapy enhances treatment efficacy while reducing the dose required for treatment, thereby preventing the risk of potential side effects and reducing the development of treatment resistance [57]. The understanding of the mechanism of the two combined drugs not only helps to explain the direct-action principle of traditional Chinese medicine on organs but also has reference significance for understanding the impact of drugs on gut health. Targeting gut barrier dysfunction is a novel strategy for preventing and treating chronic disease as an intact gut barrier could, in theory, still prevent endotoxemia-associated systemic complications, even with dysbiosis [58]. As baicalin isolated from Scutellariae radix can only be absorbed after microbiota-dependent hydrolysis to its aglycone form baicalein, this means that the biological activity exhibited by baicalein must depend on the normal function of intestinal flora [59]. Conversely, phytotherapy for treating chronic diseases could maintain the gut’s health. From our observations, we deduced that the synergistic anti-inflammatory effects of the dietary flavones BAI and GNP might be caused by the inhibition of AKT activation, which is a mechanism that is also applied in the gut. Since BAI targets HSP90, hindering its molecular chaperone cycle, AKT is unable to fold correctly and degrades. In addition, GNP directly targets the PH domain of AKT and inhibits its phosphorylation and activation.

## 5. Conclusions

In conclusion, the strong synergistic effect of BAI and GNP is attributable to the reduced expression and phosphorylation of AKT. Thus, our study elucidates the interaction between AKT and HSP90, offering new avenues for clinical application and the development of synergistic anti-inflammatory drugs.

## Data Availability

The data presented in this study are available on request from the corresponding author.
